# Petechiae and a Persistent Violaceous Nodule: A Presentation of Blastic Plasmacytoid Dendritic Cell Neoplasm to Dermatology

**DOI:** 10.1155/crdm/8628105

**Published:** 2025-03-18

**Authors:** Madison Anzelc, Christina Druskovich, Austin Cusick, Matthew Franklin

**Affiliations:** ^1^Department of Dermatology, Riverside Methodist OhioHealth Hospital, Columbus, Ohio, USA; ^2^Michigan State University College of Human Medicine, Lansing, Michigan, USA

## Abstract

Blastic plasmacytoid dendritic cell neoplasm (BPDCN) is a rare, highly aggressive CD4+ CD56+ hematopoietic malignancy. The cutaneous presentation is variable but often includes violaceous nodules. We present a rare case of BPDCN, which featured dermatological findings consisting of erythematous macules, petechiae, purpura, and a violaceous nodule. A skin biopsy and peripheral blood smear supported a diagnosis of BPDCN. With BPDCN favoring cutaneous involvement, we urge dermatologists to be aware of the possibility of a BPDCN diagnosis in patients who present with purpuric nodules and petechial skin findings, especially when it is not easily explainable by another pathology or medication.

## 1. Introduction

Blastic plasmacytoid dendritic cell neoplasm (BPDCN) is a rare, highly aggressive CD4+ CD56+ hematopoietic malignancy. From the date of diagnosis, the overall median survival is approximately 1 year [1]. BPDCN affects fewer than 1400 people throughout the United States and Europe combined per year [1]. The age of diagnosis has a bimodal distribution, with patients younger than 20 years and older than 60 years representing peak incidence demographics [2]. Males are affected approximately 4 times more often than females, which is attributable to a loss-of-function mutation on the *X* chromosome.

BPDCN is a malignant tumor derived from plasmacytoid dendritic cells. The innate functions of these cells include antigen presentation to CD4+ *T*-cells, induction of *T* helper cell differentiation, and production of type 1 interferon [3]. They are also thought to play a role in antitumor immunity [4]. Mutations in genes affecting myeloid tumor suppressors, splicing functions, RAS signaling, and epigenetic regulation have been reported in BPDCN [5].

The cutaneous presentation of BPDCN is variable but often includes one or more violaceous nodules, sometimes in addition to macular, papular, and purpuric lesions. Here, we describe an unusual presentation of BPDCN to dermatology.

## 2. Case Presentation

**Figure 1:**
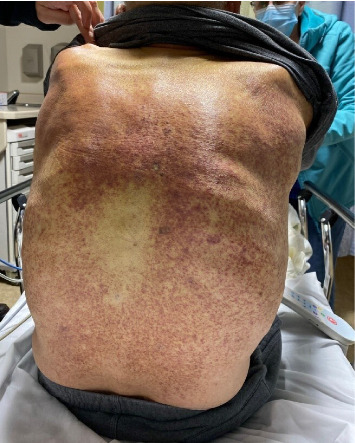
Petechiae and purpura of the back.

**Figure 2:**
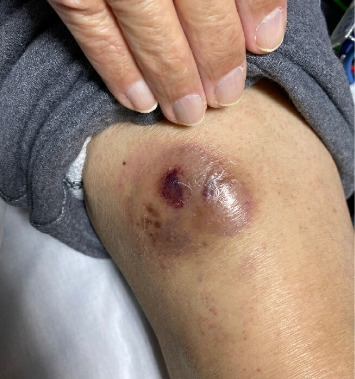
Violaceous to brown purpuric plaque of the right distal thigh.

A 76-year-old male with a history of Factor V Leiden treated with warfarin and Parkinson's disease presented to his primary care physician for a 4-month history of progressive rash. The eruption was diffuse and consisted of erythematous macules, petechiae, and purpura (Figure 1). The back, chest, and upper extremities were affected, with notable sparing of the dorsal and volar hands. The patient denied any associated pain or pruritus. During the initial evaluation, a 5 × 5 cm pearly, ecchymotic nodule involving the patient's right distal thigh was also noted (Figure 2). The patient reported that it had been present for over 6 months. The patient denied any associated symptoms but did report that the lesion may be gradually enlarging.

The primary care physician completed a workup consisting of a complete blood count, prothrombin time/international normalized ratio, and peripheral blood smear. The patient presented 1 week later to the emergency department for worsening rash, fatigue, and an increase in Parkinsonian tremors. The patient again reported no associated pruritus or pain. Physical exam was remarkable for jaundice, palatal petechiae, and conjunctival hemorrhage.

**Figure 3:**
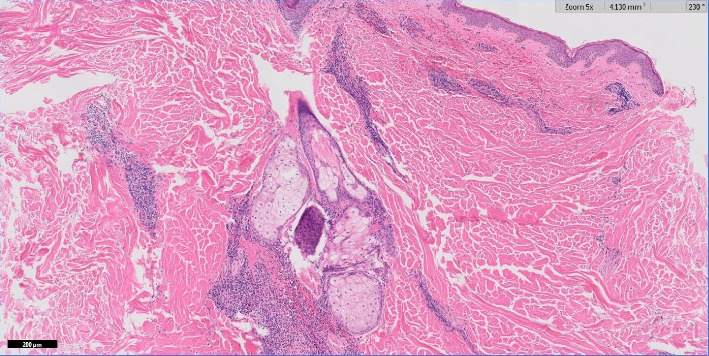
50x magnification and hematoxylin and eosin staining of the back punch biopsy with perivascular and periadnexal infiltrate.

**Figure 4:**
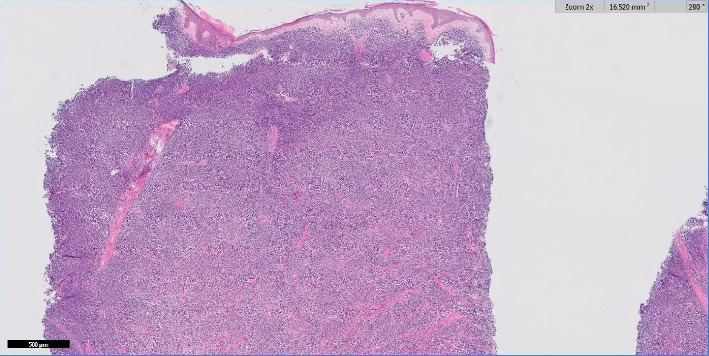
20x magnification and hematoxylin and eosin staining of right distal thigh punch biopsy demonstrating diffuse sheet-like dermal infiltrate of lymphoid cells.

**Figure 5:**
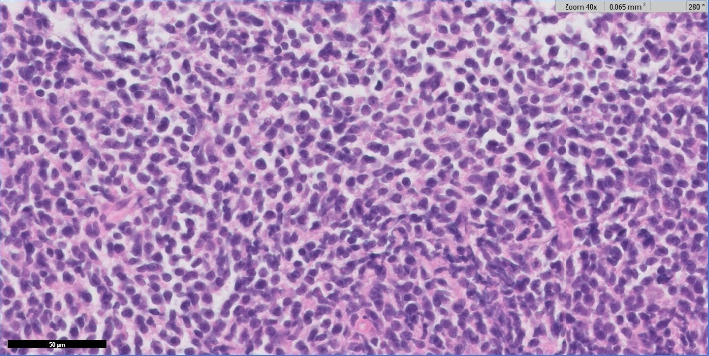
High magnification hematoxylin and eosin staining of the infiltrate demonstrates blastic cells with irregular nuclear contours, finely dispersed chromatin, and scant cytoplasm.

**Figure 6:**
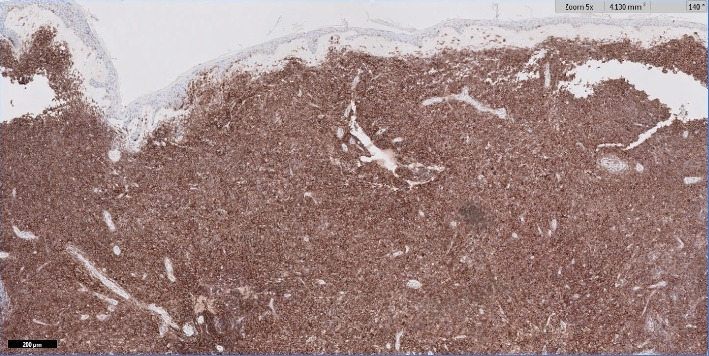
50x magnification of right distal thigh punch biopsy showing diffuse CD4 immunoreactivity.

Additional workup was ordered. A peripheral smear revealed leukopenia with circulating atypical blastoid cells that were medium in size with a high nuclear/cytoplasmic ratio, fine chromatin, and variably prominent nuclei. Peripheral flow cytometry identified an atypical CD4+ CD56+ cell population. Interventional radiology and dermatology were consulted for bone marrow and skin biopsies, respectively. A punch biopsy of the right central back demonstrated a perivascular and periadnexal lymphoid infiltrate (Figure 3). A punch biopsy specimen from the right distal thigh nodule demonstrated a diffuse dermal infiltrate of small to medium-sized lymphoid cells (Figure 4) with scant cytoplasm, irregular nuclear contours, and finely dispersed chromatin (Figure 5). Immunohistochemical staining performed on the right distal thigh biopsy revealed the lymphoid infiltrate to be immunoreactive for HLA-DR, CD2, CD4, CD7, CD56, and CD123, consistent with BPDCN (Figure 6). Immunohistochemical staining was also negative for CD34, which aids in ruling out acute myeloblastic leukemia. The diagnosis was further supported by corresponding immunophenotyping of bone marrow specimens. The patient was transferred to another institution with expertise in BPDCN.

## 3. Discussion

BPDCN is a CD4+ CD56+ hematologic neoplasm with a propensity for cutaneous involvement. It is known for its aggressive nature, predisposition for leukemic dissemination, and poor prognosis [3]. It frequently presents with cutaneous involvement, but identification and diagnosis are often delayed due to its overall rarity [6]. Reported cutaneous presentations are heterogeneous; however, the most common initial presentation is cutaneous nodules. Some other common cutaneous manifestations include a mixture of infiltrated tumors or nodules, violaceous bruise-like macules, and other nonspecific lesions [3, 7]. A cohort study found that almost half of patients with BPDCN initially presented with one or two isolated cutaneous nodules. The most commonly affected location is the face or scalp, followed by the lower limb, trunk, and upper limb. The majority of patients presenting with bruise-like macules demonstrated the involvement of 1 or 2 body areas with several lesions. A minority of the “bruise-like” patch patients had mucosal involvement or a single lesion [7]. Although there appears to be a classic cutaneous presentation, multiple case reports attest that BPDCN can also mimic other pathologies. More specifically, case reports have shown BPDCN being misdiagnosed as an invading ductal carcinoma and as rheumatic fever in a child [8, 9]. We opine that any elderly patient presenting with hemorrhagic to purpuric lesion(s) ought to raise early consideration of this neoplasm as, despite its rarity and protean clinical manifestations, its aggressive nature and poor prognosis earn it a high position on the differential diagnosis.

Tissue biopsy is instrumental for diagnosis and should be performed as soon as BPDCN is suspected. The biopsy must include the deep dermis and subcutaneous fat, and it is crucial to notify the pathologist that BPDCN is on the differential since infrequently used immunohistochemical stains are essential to make the diagnosis [10].

The prognosis of BPDCN is poor, with a median survival of 12–14 months [11]. New therapies are currently being investigated through clinical trials or have recently been approved [12, 13]. The aggressive nature of BPDCN makes it a diagnosis not to be missed; dermatologists need to be familiar with both the common presenting cutaneous manifestations and more atypical presentations.

## Data Availability

Data sharing is not applicable to this article as no new data were created or analyzed in this study.
